# Gender differences in the relationship between serum uric acid and the long-term prognosis in heart failure: a nationwide study

**DOI:** 10.1186/s12933-024-02214-1

**Published:** 2024-04-18

**Authors:** Kang Fu, Congyi Cheng, Cong Su, Junlin Teng, Lei Qiao, Jie Xiao, Xiaoping Ji, Huixia Lu, Wenqiang Chen

**Affiliations:** 1 State Key Laboratory for Innovation and Transformation of Luobing Theory, Key Laboratory of Cardiovascular Remodeling and Function Research, Chinese Ministry of Education, Chinese National Health Commission and Chinese Academy of Medical Sciences; Department of Cardiology, Qilu Hospital of Shandong University, Jinan, China; 2 Department of Critical Care Medicine, Qilu Hospital of Shandong University, Jinan, Shandong 250012 China

**Keywords:** Gender medicine, Uric acid, Heart failure, Prognosis

## Abstract

**Background:**

Serum uric acid (SUA) is an important pathogenetic and prognostic factor for heart failure (HF). Gender differences are apparent in HF. Furthermore, gender differences also exist in the association between SUA and prognosis in various cardiovascular diseases. However, the gender difference for SUA in the prediction of long-term prognosis in HF is still ambiguous.

**Methods:**

A total of 1593 HF patients (897 men, 696 women) from the National Health and Nutrition Examination Survey (NHANES) 1999–2018 cycle were enrolled in our final analysis. Participants were categorized according to gender-specific SUA tertile. We assessed the association between SUA and long-term prognosis of HF patients, defined as all-cause mortality and cardiovascular mortality, in different genders via Kaplan–Meier curve analysis, Cox proportional hazard model, and Fine-Gray competing risk model. The restricted cubic spline (RCS) was performed to investigate the dose-response relationship between SUA and outcomes.

**Results:**

Gender differences exist in demographic characteristics, clinical parameters, laboratory tests, and medication of HF patients. After a median follow-up of 127 months (95% CI 120–134 months), there were 853 all-cause deaths (493 events in men, 360 events in women) and 361 cardiovascular deaths (206 events in men, 155 events in women). Kaplan-Meier analysis showed that SUA had gender difference in the prediction of cardiovascular mortality (Log-rank *p* < 0.001, for male, Log-rank *p* = 0.150, for female), but not in all-cause mortality. Multivariate Cox regression analysis revealed that elevated SUA levels were associated with higher all-cause mortality and cardiovascular mortality in men (HR 1.11, 95% CI 1.05-1.18, *p* < 0.001, for all-cause death; HR 1.18, 95% CI 1.09-1.28, *p* < 0.001, for cardiovascular death), but not in women (HR 1.05, 95% CI 0.98-1.12, *p* = 0.186, for all-cause death; HR 1.01, 95% CI 0.91-1.12, *p* = 0.902, for cardiovascular death). Even using non-cardiovascular death as a competitive risk, adjusted Fine-Gray model also illustrated that SUA was an independent predictor of cardiovascular death in men (SHR 1.17, 95% CI 1.08-1.27, *p* < 0.001), but not in women (SHR 0.98, 95% CI 0.87 − 1.10, *p* = 0.690).

**Conclusions:**

Gender differences in the association between SUA and long-term prognosis of HF existed. SUA was an independent prognostic predictor for long-term outcomes of HF in men, but not in women.

## Introduction

Numerous epidemiological and clinical investigations have revealed that serum uric acid (SUA), the end-product of purine metabolism, plays a vital pathogenetic role in heart failure (HF) [1–3]. Hyperuricemia is a well-recognized risk factor for HF, and an elevated SUA concentration is associated with poor prognosis in patients with HF [4–6]. However, the underlying pathogenesis mechanism of SUA in HF is still unclear. The increase in oxidative stress, activation of pro-inflammatory pathways, and impairment of fatty acid metabolism caused by elevated SUA levels may be potential molecular mechanisms [7–9].

The sex differences of cardiovascular risk factors and outcomes are noticeable [10, 11]. For HF patients, gender differences are also apparent. Previous studies have demonstrated that female HF patients have specific etiologies, unique predispositions, peculiar spectra of HF phenotypes, and more favorable survival and prognosis [12, 13]. There are also gender differences in SUA concentrations and prognostic value for various cardiovascular diseases. Generally, male patients exhibit higher SUA concentrations than female patients [14]. The associations between SUA and the prognosis of several cardiovascular diseases, including acute coronary syndrome, metabolic syndrome, left ventricular hypertrophy (LVH), and atrial fibrillation, are more tightly and pronounced in women [15–18].

Despite the gender difference in the prognostic implication of SUA in numerous cardiovascular diseases, studies reporting the gender differences in the association between SUA and the prognosis of HF are limited. The gender difference in the association between SUA and long-term prognosis in patients with HF is still ambiguous and controversial. Thus, we conducted a post-hoc analysis of a nationwide longitudinal cohort study to explore how gender-differences can impact the prognostic value of SUA for the long-term prognosis of HF.

## Methods

### Data source and study population

The study was a post-hoc analysis of the National Health and Nutrition Examination Survey (NHANES), which was a nationally representative health survey study recruiting participants from the noninstitutionalized civilian population in the United States. NHANES was conducted by the National Center for Health Statistics (NCHS). The study methodology and data collecting procedure had been previously reported and published [19, 20]. Ethical approval of NHANES was obtained through the institutional ethics review board of the NCHS and written informed consent was obtained for all participants (the NCHS Ethics Review Board (ERB) approval was searchable online). All data were publicly accessible online (https://www.cdc.gov/nchs/nhanes/index.htm).

The study initially screened 102,956 participants from the NHANES 1999–2018 cycle. The inclusion criteria included: (i) self-reported HF, which was defined by answering yes to the question regarding their medical history: “Has a doctor or health professional ever told you that you had congestive HF?”; (ii) age≥20 years old; (iii) retrievable SUA data. The exclusion criteria include: (i) age less than 20 years old; (ii)incomplete data of SUA; and (iii) incomplete mortality data. After exclusion, a total of 1593 HF patients were enrolled in our final analysis.

### SUA measurement

The blood sample obtained from participants were stored and measured according to the laboratory procedure manual of the NHANES. The colorimetric method was applied to detect SUA. A Beckman UniCel DxC800 Synchron analyser was used for SUA measurements in the NHANES 1999–2016 cycle, while a Roche Cobas 6000 analyser was applied in the measurement of SUA in the NHANES 2017–2018 cycle. More detailed operation protocol and procedures could be found in the official website.

### Demographic and clinical data

Demographic and clinical data, including (i) demographic characteristics, including age, gender, race-ethnicity (categorized as non-Hispanic white, non-Hispanic black, Mexican American, or other), smoking history, and drinking history; (ii) physical examination, including body mass index (BMI), heart rate, systolic blood pressure (SBP), and diastolic blood pressure (DBP); (iii) comorbidities, including hypertension, diabetes mellitus, coronary heart disease (CHD), and stroke; (iv) laboratory tests, including white blood cell, hemoglobin, albumin, hemoglobin A1c (HbA1c), total cholesterol, triglyceride, serum glucose, serum creatinine, serum sodium, serum potassium, and serum chloride; (v) medication, including angiotensin-converting inhibitor/ angiotensin II receptor blocker (ACEI/ARB), beta blockers, mineralocorticoid-receptor antagonist, diuretics, and urate-lowering drugs, were collected. The estimated glomerular filtration rate (eGFR) was calculated via the Chronic Kidney Disease Epidemiology Collaboration creatinine 2021 (CKD-EPI 2021) equation. The eGFR = 142×min(serum creatinine/κ, 1)^α^×max(serum creatinine/κ, 1)^−1.200^ × 0.9938^age^×(1.012[if female]) (annotations: κ is 0.7 for women and 0.9 for men; α is -0.241 for women and − 0.302 for men). Hypertension was defined as self-reported hypertension to corresponding questionnaire, or SBP ≥ 140 mmHg, or DBP ≥ 90 mmHg, or taking antihypertensive drugs. Diabetes mellitus was defined as self-reported diabetes, or HbA1c≥6.5%, or using of diabetes medication. CAD and stroke were confirmed by self-reported diagnosis.

## Outcomes

The study outcomes include all-cause death and cardiovascular death. The mortality data were extracted from the National Death Index were linked to NHANES. The Linked Mortality Data Files with mortality follow-up data have been updated through December 31, 2019. Each decreased participant was ascertained by the death certificate records. The cause of death was determined to the 10th version of the International Classification of Disease (ICD-10). The cardiovascular death was defined as deaths due to heart disease (ICD codes I00-I09, I11, I13, 120-I25, and I26-I51) or cerebrovascular disease (ICD codes I60-I69).

### Statistical analysis

The statistical analysis was performed according with the guidance of NCHS for the complex design of the NHANES. For demographic and clinical covariates, continuous variables were presented as medians with interquartile ranges, and categorical variables were described as frequencies and percentages. Mann-Whitney U test was performed for continuous variables, while the Kruskal–Wallis H test, Chi-square test or Fisher’s exact probability tests were conducted to investigate the presence of differences between categorical variables, as appropriate. Missing covariates were handled using multiple imputation.

We used the G*power software (version 3.1) to examine the statistical power of our study sample size. The ANOVA test revealed that the power of the study was higher than 0.90 for both genders, with the effect size of 0.4, α err prob of 0.05. The values indicated that our sample size was sufficient. To evaluate gender differences in the association between SUA and the long-term prognosis in patients with HF, participants were categorized as different subgroups according to gender-specific SUA tertiles. Kaplan–Meier curve analysis and log-rank test were conducted to evaluate the overall survival rate. The relationship between SUA and all-cause mortality/cardiovascular mortality was assessed using the Cox proportional hazard model. After univariable analysis, covariates with statistical significance (*P*<0.05) would be further enrolled in the adjusted model via multivariable Cox regression analysis. For the adjusted model in the association of SUA and all-cause mortality, covariates including age, race, diabetes, stroke, drinking history, BMI, diastolic BP, WBC, hemoglobin, albumin, total cholesterol, eGFR, serum potassium, serum sodium, serum chloride, prescription of beta-blockers, prescription of MRA, prescription of diuretics, and prescription of urate-lowering agents, were adjusted. For the adjusted model in the association of SUA and cardiovascular mortality, covariates including age, race, stroke, BMI, diastolic BP, hemoglobin, triglyceride, albumin, eGFR, serum sodium, serum potassium, serum chloride, prescription of beta-blockers, prescription of MRA, prescription of diuretics, and prescription of urate-lowering agents, were adjusted to eliminate potential confounders. Hazard ratios (HRs) and 95% confidence intervals (CIs) were estimated. Furthermore, to comprehensively assess the association between SUA and cardiovascular deaths, we used the Fine-Gray test to analyze non-cardiovascular death as a competitive risk. The sub-distribution hazard ratio (SHR) and 95% CI was calculated by Fine-Gray competing risk model. The restricted cubic spline (RCS) was performed to investigate the dose-response relationship (linear or non-linear) between SUA and all-cause mortality/cardiovascular mortality. In order to ensure the best fit of RCS, the number of knots for the RCS regression was four to meet the smallest Akaike information criterion. A two-tailed p-value less than 0.05 was regarded as statistically significant.

The data were analyzed via IBM SPSS Statistics version 25, 2017 (IBM, Armonk, New York) and R (version 4.1.0) software.

## Results

### Baseline clinical characteristics

After exclusion, a total of 1593 patients with HF from the NHANES were enrolled in our final analysis. The baseline demographic, clinical and biochemical characteristics of both men and women stratified by gender-specific SUA tertiles were illustrated in Tables 1 and 2. The participants of our investigation included 897 (56.3%) men and 696 (43.7%) women. For the demographic characteristics, the age distribution by SUA tertiles varied between men and women patients. The positive correlation of SUA tertiles and age was only observed in women (*p* < 0.001) rather in men (*p* = 0.326). Furthermore, the proportion of patients with diabetes increased with SUA tertiles only in women (*p* = 0.005) but not in men (*p* = 0.681), while the proportion of patients with hypertension increased with SUA tertiles in both genders. For both men and women, a higher SUA tertile was associated with increased BMI. The gender differences were also observed in laboratory tests. Serum HbA1c, glucose, and potassium increased with SUA tertiles only in women (HbA1c, *p* < 0.001; serum glucose, *p* = 0.001; and serum potassium, *p* < 0.001, respectively) but not in men. In contrast, higher tertiles of SUA correlated with decreased hemoglobin and decreased eGFR in both male and female patients (*p* < 0.05, both). For the medication, the prescription rate of ACEI/ARB, betablockers, MRA, and diuretics were higher in patients with higher SUA tertiles, for both male and female patients.

**Table 1:** Baseline demographic and clinical characteristics in men

	All (N=897)	Tertile 1 (N=306)	Tertile 2 (N=299)	Tertile 3 (N=292)
Uric acid (mg/dL)	6.6 (5.4-7.9)	<5.9	5.9-7.4	>7.4
Age, years	70 (61-78)	70 (60-78)	68 (60-77)	70 (61-78)
Body mass index, kg/m^2^	29.6 (25.9-34.0)	28.1 (24.2-32.3)	30.4 (26.4-34.7) ^†^	30.5 (26.4-35.1) ^†^
Systolic BP, mmHg	128 (115-142)	128 (116-142)	128 (115-142)	126 (113-144)
Diastolic BP, mmHg	68 (58-78)	69 (58-77)	70 (60-78)	66 (58-77)
Heart rate, bpm	70 (62-78)	70 (60-76)	70 (60-78)	69 (62-78)
Race, n (%)				
Non-Hispanic White	522 (58.2)	173 (56.5)	178 (59.5)	171 (58.6)
No-Hispanic Black	179 (20.0)	54 (17.6)	47 (15.7)	78 (26.7) ^†‡^
Mexican American	93 (10.4)	37 (12.1)	38 (12.7)	18 (6.2) ^†‡^
Other	103 (11.4)	42 (13.8)	36 (12.1)	25 (8.5) ^†^
History of smoking, n (%)	643 (71.7)	215 (70.3)	218 (72.9)	210 (71.9)
History of drinking, n (%)	691 (77.0)	227 (74.2)	241 (80.6)	223 (76.4)
Hypertension, n (%)	721 (80.4)	231 (75.5)	241 (80.6)	249 (85.3) ^†^
Diabetes mellitus, n (%)	387 (43.1)	134 (43.8)	123 (41.1)	130 (44.5)
Coronary heart disease, n (%)	432 (48.2)	148 (48.4)	153 (51.2)	131 (44.9)
Stroke, n (%)	184 (20.5)	72 (23.5)	54 (18.1)	58 (19.9)
Laboratory tests				
WBC, 10^3^/uL	7.3 (6.1-8.8)	7.3 (6.0-8.5)	7.3 (6.0-8.8)	7.3 (6.1-8.9)
Hemoglobin, g/dL	14.0 (12.5-15.0)	14.1 (12.6-15.1)	14.1 (12.8-15.0)	13.7 (12.1-14.9) ^‡^
Albumin, g/L	41.0 (39.0-43.0)	41.0 (38.0-44.0)	41.0 (39.0-43.0)	41.0 (39.0-43.0)
HbA1c, %	5.9 (5.5-6.6)	5.9 (5.4-6.7)	5.9 (5.5-6.5)	6.0 (5.5-6.6)
Total cholesterol, mmol/L	4.3 (3.6-5.1)	4.3 (3.6-5.0)	4.4 (3.6-5.2)	4.4 (3.6-5.3)
Triglycerides, mg/dL	135.0 (91.5-200.5)	128.0 (84.0-192.5)	143.0 (97.0-202.0)	139.0 (95.0-205.8)
Serum glucose, mg/dL	102.0 (92.0-128.0)	102.0 (91.0-135.0)	100.0 (91.0-124.0)	104.0 (93.0-125.0)
eGFR, mL/min/1.73m^2^	67.5 (50.2-88.2)	76.8 (59.3-94.7)	69.5 (53.3-88.8) ^†^	55.8 (40.4-71.2) ^†‡^
Serum sodium, mmol/L	139.0 (138.0-141.0)	139.0 (137.0-141.0)	139.0 (138.0-141.0)	139.0 (138.0-141.0)
Serum potassium, mmol/L	4.2 (3.9-4.5)	4.2 (4.0-4.5)	4.1 (3.9-4.4) ^†^	4.2 (3.9-4.5) ^‡^
Serum chloride, mmol/L	103.0 (100.0-105.0)	103.0 (100.0-105.0)	103.0 (101.0-105.0)	102.0 (100.0-105.0)
Medication				
ACEI/ARB, n (%)	504 (56.2)	145 (47.4)	167 (55.9) ^†^	192 (65.8) ^†‡^
Beta blockers, n (%)	534 (59.5)	169 (55.2)	171 (57.2)	194 (66.4) ^†‡^
MRA, n (%)	64 (7.1)	9 (2.9)	19 (6.4) ^†^	36 (12.3) ^†‡^
Diuretics, n (%)	431 (48.0)	89 (29.1)	135 (45.2) ^†^	207 (70.9) ^†‡^
Urate-lowering agents, n (%)	78 (8.7)	37 (12.1)	27 (9.0)	14 (4.8) ^†‡^

Values are presented as median (interquartile range), or n (%). For *P*-value, ^†^ indicates p value < 0.05 versus Tertile 1, ^‡^ indicates p value < 0.05 versus Tertile 2

Abbreviations: ACE-I, angiotensin-converting inhibitor; ARB, angiotensin II receptor blocker; BP, blood pressure; eGFR, the rate of estimated glomerular filtration rate; Hb1Ac, hemoglobin A1c; MRA, mineralocorticoid-receptor antagonist; WBC, white blood cells

**Table 2:** Baseline demographic and clinical characteristics in women

	All (N=696)	Tertile 1 (N=223)	Tertile 2 (N=235)	Tertile 3 (N=238)
Uric acid (mg/dL)	5.9 (4.8-7.1)	<5.1	5.1-6.5	>6.5
Age, years	71 (60-80)	66 (51-78)	71 (61-80) ^†^	74 (64-80) ^†^
Body mass index, kg/m^2^	30.6 (25.9-37.8)	28.6 (25.1-34.0)	31.8 (26.2-37.9) ^†^	31.7 (27.3-40.3) ^†^
Systolic BP, mmHg	134 (118-150)	134 (118-148)	136 (119-152)	132 (116-146)
Diastolic BP, mmHg	65 (56-74)	66 (58-74)	66 (58-76)	64 (54-72) ^† ‡^
Heart rate, bpm	70 (62-80)	70 (64-80)	70 (62-78)	72 (62-80)
Race, n (%)				
Non-Hispanic White	348 (50.0)	113 (50.7)	114 (48.5)	121 (50.8)
No-Hispanic Black	188 (27.0)	49 (22.0)	63 (26.8)	76 (31.9) ^†^
Mexican American	81 (11.6)	32 (14.3)	35 (14.9)	14 (5.9) ^†‡^
Other	79 (11.4)	29 (13.0)	23 (9.8)	27 (11.4)
History of smoking, n (%)	333 (47.8)	104 (46.6)	122 (51.9)	107 (45.0)
History of drinking, n (%)	306 (44.0)	100 (44.8)	109 (46.4)	97 (40.8)
Hypertension, n (%)	594 (85.3)	178 (79.8)	206 (87.7) ^†^	210 (88.2) ^†^
Diabetes mellitus, n (%)	312 (44.8)	90 (40.4)	95 (40.4)	127 (53.4) ^†‡^
Coronary heart disease, n (%)	209 (30.0)	69 (30.9)	69 (29.4)	71 (29.8)
Stroke, n (%)	148 (21.3)	52 (23.3)	44 (18.7)	52 (21.8)
Laboratory tests				
WBC, 10^3^/uL	7.2 (5.9-8.8)	7.1 (5.9-8.4)	7.1 (5.9-9.0)	7.4 (6.1-8.8)
Hemoglobin, g/dL	12.7 (11.4-13.7)	12.9 (11.4-13.7)	12.9 (11.9-13.9)	12.3 (11.3-13.5) ^† ‡^
Albumin, g/L	40.0 (38.0-42.0)	40.0 (38.0-42.0)	40.0 (38.0-42.0)	40.0 (38.0-42.0)
HbA1c, %	5.9 (5.5-6.7)	5.8 (5.4-6.5)	5.9 (5.6-6.6) ^†^	6.1 (5.6-7.1) ^† ‡^
Total cholesterol, mmol/L	4.8 (4.0-5.6)	4.9 (3.9-5.8)	5.0 (4.2-5.8)	4.6 (3.8-5.3) ^‡^
Triglyceride, mg/dL	135.0 (95.0-195.0)	122.0 (82.0-186.0)	141.0 (102.0-194.0) ^†^	137.5 (104.0-202.5) ^†^
Serum glucose, mg/dL	102.0 (91.3-129.0)	99.0 (88.0-120.0)	100.0 (90.0-123.0)	107.0 (94.0-140.3) ^† ‡^
eGFR, mL/min/1.73m^2^	77.1 (49.7-99.9)	94.1 (69.9-109.8)	79.3 (59.6-98.4) ^†^	53.9 (36.7-81.4) ^† ‡^
Serum sodium, mmol/L	139.0 (137.9-141.0)	139.0 (137.0-141.0)	140.0 (138.0-142.0) ^†^	139.6 (138.0-141.8) ^†^
Serum potassium, mmol/L	4.1 (3.8-4.4)	4.0 (3.7-4.3)	4.1 (3.8-4.3) ^†^	4.2 (3.9-4.5) ^†^
Serum chloride, mmol/L	102.0 (100.0-105.0)	102.0 (99.0-105.0)	103.0 (100.0-105.0)	102.0 (100.0-105.0)
Medication				
ACEI/ARB, n (%)	373 (53.6)	103 (46.2)	134 (57.0) ^†^	136 (57.1) ^†^
Beta blockers, n (%)	357 (51.3)	91 (40.8)	125 (53.2) ^†^	141 (59.2) ^†^
MRA, n (%)	62 (8.9)	9 (4.0)	20 (8.5) ^†^	33 (13.9) ^†^
Diuretics, n (%)	378 (54.3)	81 (36.3)	121 (51.5) ^†^	176 (73.9) ^†‡^
Urate-lowering agents, n (%)	28 (4.0)	10 (4.5)	6 (2.6)	12 (5.0)

Values are presented as median (interquartile range), or n (%). For *P*-value, ^†^ indicates p value < 0.05 versus Tertile 1, ^‡^ indicates p value < 0.05 versus Tertile 2

Abbreviations: ACE-I, angiotensin-converting inhibitor; ARB, angiotensin II receptor blocker; BP, blood pressure; eGFR, the rate of estimated glomerular filtration rate; Hb1Ac, hemoglobin A1c; MRA, mineralocorticoid-receptor antagonist; WBC, white blood cells

### SUA levels and clinical outcomes in different genders

**Fig. 1:**
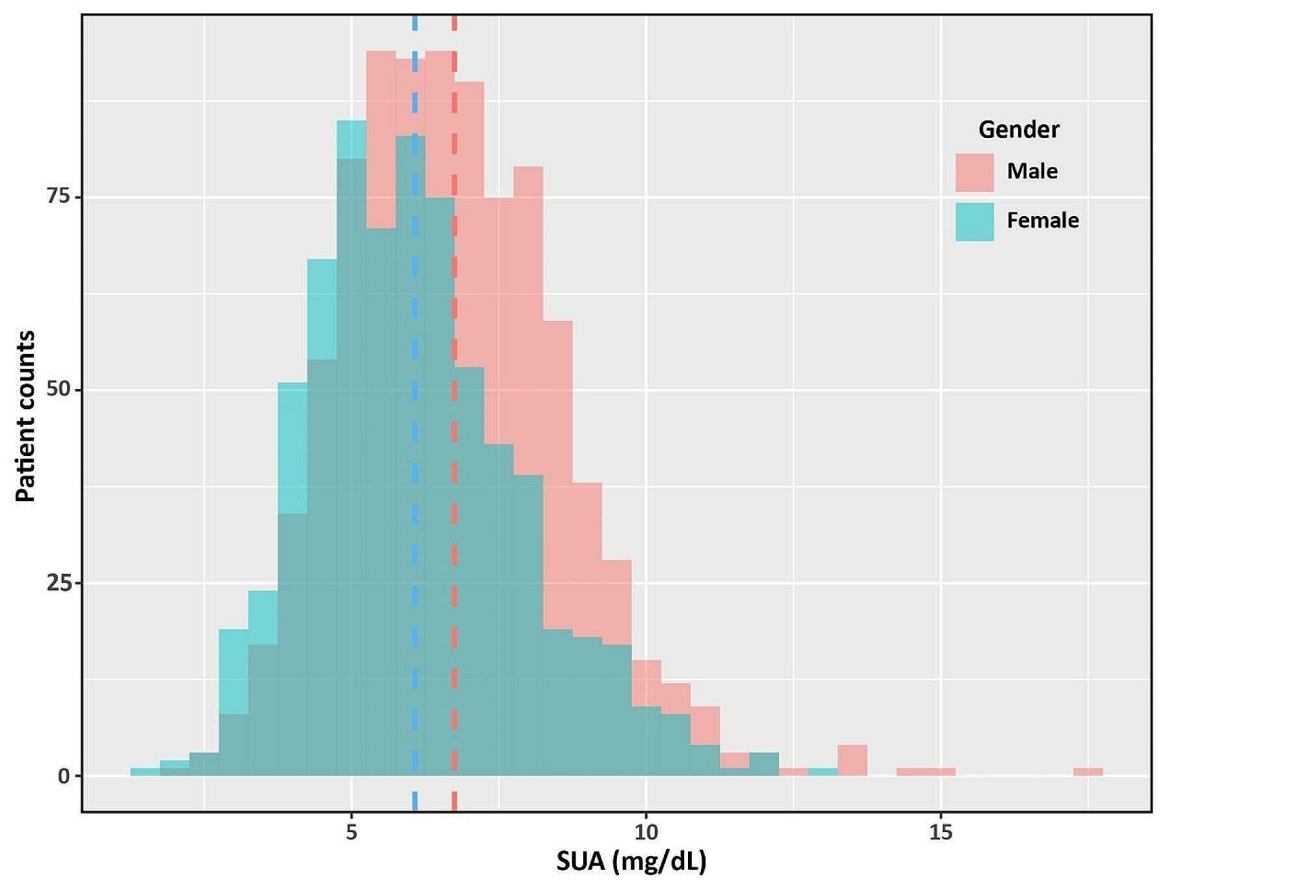
Histogram of SUA in male and female HF patients

As shown by histogram (Fig. 1), the median level of SUA in women was significantly lower than that in men (5.9 mg/dL, IQR 4.8-7.1 mg/dL vs. 6.6 mg/dL, IQR 5.4-7.9 mg/dL, *p* < 0.001). During a median follow-up of 127months (95% CI 120–134 months), there were 853 all-cause deaths (493 events in men, 360 events in women) and 361 cardiovascular deaths (206 events in men, 155 events in women). There was no significantly gender difference regarding to all-cause and cardiovascular mortality. The occurrence of all-cause death (55.0% vs. 51.7%, *p* = 0.199) and cardiovascular death (23.0% vs. 22.3%, *p* = 0.742) had no statistical difference between men and women. When analysis of long-term outcomes according to SUA tertiles and gender, the all-cause mortality was higher in patients with a higher SUA tertile in both genders (men, *p* = 0.001; women, *p* = 0.031). While the positive association between SUA and cardiovascular death was only observed in men (*p* = 0.001), but not in women (*p* = 0.691) (Table 3).

**Table 3:** Proportion of all-cause and cardiovascular events in patients according to gender and SUA tertiles

Whole population (N=1593)	Tertile 1, SUA: <5.5 mg/dL	Tertile 2, SUA: 5.6-7.0 mg/dL	Tertile 3, SUA: >7.0 mg/dL	p value
All-cause death, n (%)	262 (50.3)	256 (48.9)	335 (60.9)	<0.001
Cardiovascular death, n (%)	111 (21.3)	99 (18.9)	151 (27.5)	0.003
**Men (**N**=897, 56.3%)**	Tertile 1, SUA: <5.9 mg/dL	Tertile 2, SUA: 5.9-7.4 mg/dL	Tertile 3, SUA: >7.4 mg/dL	*p* value
All-cause death, n (%)	150 (49.0)	156 (52.2)	187 (64.0)	0.001
Cardiovascular death, n (%)	59 (19.3)	57 (19.1)	90 (30.8)	0.001
**Women (**N**=696, 43.7%)**	Tertile 1, SUA: <5.1 mg/dL	Tertile 2, SUA: 5.1-6.5 mg/dL	Tertile 3, SUA: >6.5 mg/dL	*p* value
All-cause death, n (%)	101 (45.3)	122 (51.9)	137 (57.6)	0.031
Cardiovascular death, n (%)	46 (20.6)	52 (22.1)	57 (23.9)	0.691

Values are presented as n (%)

Abbreviation: SUA, serum uric acid

### Role of SUA in prediction of long-term outcomes in different genders

**Fig. 2:**
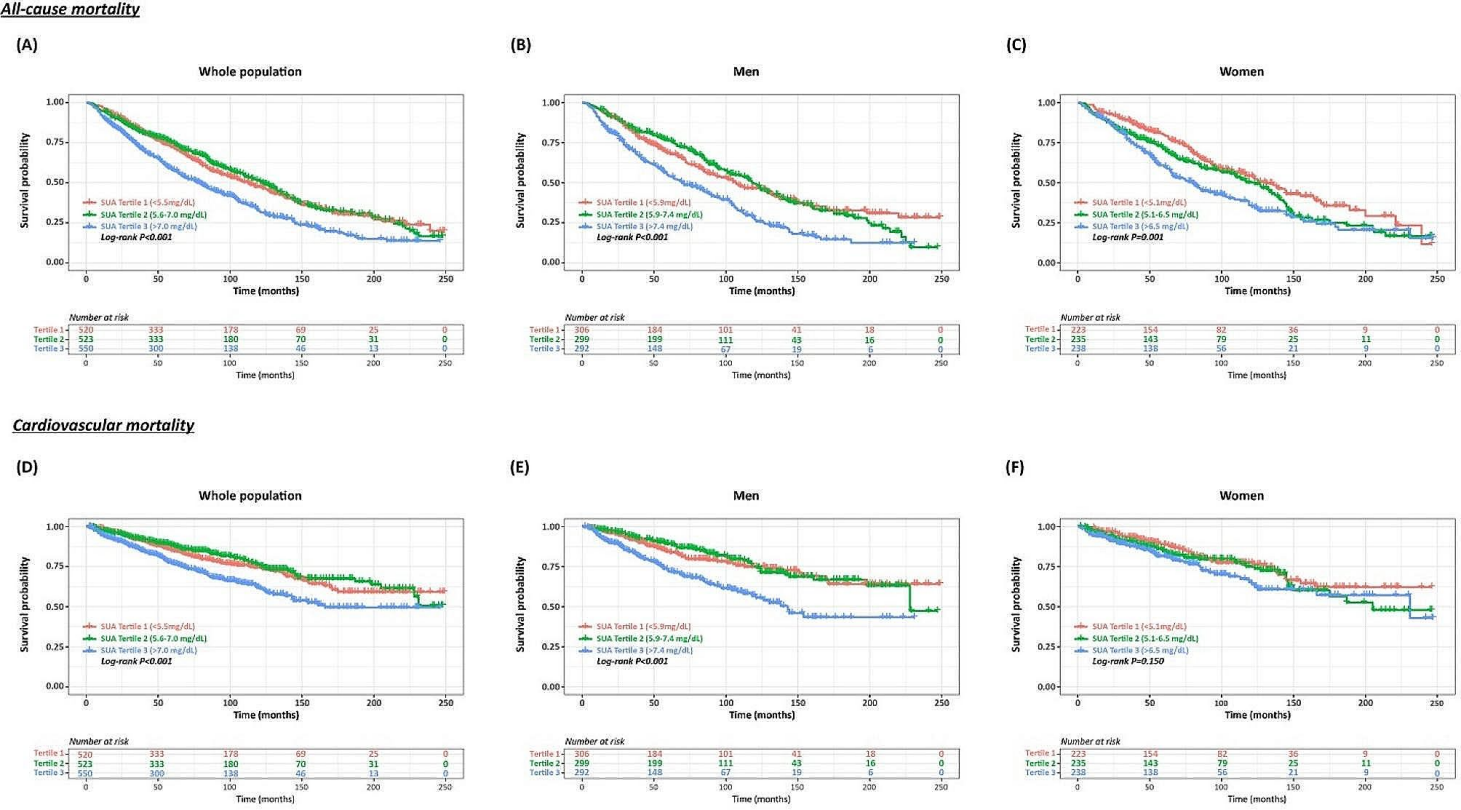
Kaplan-Meier curves for all-cause mortality and cardiovascular mortality according to gender-specific SUA tertiles

Kaplan-Meier analysis was conducted to explore the gender differences in the association between SUA and the long-term prognosis in patients with HF. There was no significantly gender difference for SUA in the prediction of long-term all-cause mortality. In the whole population and both genders, individuals with SUA in tertile 3 had significantly higher all-cause mortality than those with SUA in tertile 1–2 (Log-rank *p* < 0.010, both, Fig. 2A-C). Furthermore, for patients with SUA in tertile 1 and 2, SUA showed poor discrimination ability for all-cause mortality in both men (Log-rank *p* = 0.898) and women (Log-rank *p* = 0.065). When it comes to cardiovascular mortality, gender difference emerged. As shown in Fig. 2D-F, male patients with SUA in tertile 3 was associated with highest cumulative incidence of cardiovascular deaths (Log-rank *p* < 0.001). While for female patients, SUA performed poorly in prediction of cardiovascular mortality (Log-rank *p* = 0.150).

The prognostic value of SUA on long-term outcomes of CHF was also analyzed by Cox regression analysis. On univariable Cox regression analysis, SUA was significantly associated with all-cause mortality in the whole population (HR 1.15, 95% CI 1.11-1.19, *p* < 0.001) and both genders (HR 1.14, 95% CI 1.09-1.20, *p* < 0.001, in men; HR 1.16, 95% CI 1.10-1.23, *p* < 0.001, in women) (Table 4). However, after adjusted by covariates via multivariable Cox regression analysis, SUA (1 mg/dL) was a significant predictor of overall mortality only in men (HR 1.11, 95% CI 1.05-1.18, *p* < 0.001) but not in women (HR 1.05, 95% CI 0.98-1.12, *p* = 0.186).

**Table 4:** Association of SUA and all-cause mortality via Cox regression analysis

	Unadjusted model		Adjusted model	
	HR (95% CI)	*p* value	HR (95% CI)	*p* value
**Whole population (N=1593)**				
Continuous per 1 unit increase	1.15 (1.11-1.19)	<0.001	1.09 (1.05-1.14)	<0.001
Tertile 1 (<5.5 mg/dL)	Reference	--	Reference	--
Tertile 2 (5.6-7.0 mg/dL)	0.96 (0.81-1.14)	0.657	0.94 (0.78-1.12)	0.474
Tertile 3 (>7.0 mg/dL)	1.52 (1.29-1.78)	<0.001	1.28 (1.06-1.54)	0.009
**Men (N=897, 56.3%)**				
Continuous per 1 unit increase	1.14 (1.09-1.20)	<0.001	1.11 (1.05-1.18)	<0.001
Tertile 1 (<5.9 mg/dL)	Reference	--	Reference	--
Tertile 2 (5.9-7.4 mg/dL)	0.99 (0.79-1.24)	0.914	1.05 (0.82-1.33)	0.720
Tertile 3 (>7.4 mg/dL)	1.71 (1.38-2.12)	<0.001	1.49 (1.17-1.91)	0.001
**Women (N=696, 43.7%)**				
Continuous per 1 unit increase	1.16 (1.10-1.23)	<0.001	1.05 (0.98-1.12)	0.186
Tertile 1 (<5.1 mg/dL)	Reference	--	Reference	--
Tertile 2 (5.1-6.5 mg/dL)	1.28 (0.99-1.67)	0.064	0.97 (0.73-1.28)	0.834
Tertile 3 (>6.5 mg/dL)	1.64 (1.27-2.12)	<0.001	1.03 (0.77-1.39)	0.836

The multivariate Cox regression analysis was adjusted for age, race, diabetes, stroke, drinking history, BMI, diastolic BP, WBC, hemoglobin, albumin, total cholesterol, eGFR, serum potassium, serum sodium, serum chloride, prescription of beta-blockers, prescription of MRA, prescription of diuretics, and prescription of urate-lowering agents

Abbreviation: BMI, body mass index; BP, blood pressure, eGFR, the rate of estimated glomerular filtration rate; WBC, white blood cell; MRA, mineralocorticoid-receptor antagonist; HR, hazard ratio; CI, confidence interval

For cardiovascular mortality, similar gender difference was also observed. On crude Cox analysis, SUA was an importantly prognostic factor for the incidence of cardiovascular deaths in overall patients (HR 1.19, 95% CI 1.13-1.25, *p* < 0.001) and both genders (HR 1.21, 95% CI 1.13-1.29, *p* < 0.001, in men; HR 1.15, 95% CI 1.06-1.26, *p* = 0.001, in women) (Table 5). After adjusted by multivariate Cox analysis, the association of SUA and cardiovascular mortality was only detected in men (HR 1.18, 95% CI 1.09-1.28, *p* < 0.001) but not in women (HR 1.01, 95% CI 0.91-1.12, *p* = 0.902).

**Table 5:** Association of SUA and cardiovascular mortality via Cox regression analysis

	Non-adjusted model		Adjusted model	
	HR (95% CI)	*p* value	HR (95% CI)	*p* value
**Whole population (N=1593)**				
Continuous per 1 unit increase	1.19 (1.13-1.25)	<0.001	1.12 (1.05-1.19)	<0.001
Tertile 1 (<5.5 mg/dL)	Reference	--	Reference	--
Tertile 2 (5.5-7.0 mg/dL)	0.88 (0.67-1.16)	0.365	0.84 (0.64-1.11)	0.225
Tertile 3 (>7.0 mg/dL)	1.58 (1.24-2.02)	<0.001	1.27 (0.96-1.68)	0.097
**Men (N=897, 56.3%)**				
Continuous per 1 unit increase	1.21 (1.13-1.29)	<0.001	1.18 (1.09-1.28)	<0.001
Tertile 1 (<5.9 mg/dL)	Reference	--	Reference	--
Tertile 2 (5.9-7.4 mg/dL)	0.92 (0.64-1.32)	0.645	0.96 (0.65-1.40)	0.817
Tertile 3 (>7.4 mg/dL)	2.02 (1.46-2.81)	<0.001	1.76 (1.21-2.57)	0.003
**Women (N=696, 43.7%)**				
Continuous per 1 unit increase	1.15 (1.06-1.26)	0.001	1.01 (0.91-1.12)	0.902
Tertile 1 (<5.1 mg/dL)	Reference	--	Reference	--
Tertile 2 (5.1-6.5 mg/dL)	1.19 (0.80-1.77)	0.387	0.80 (0.52-1.22)	0.300
Tertile 3 (>6.5 mg/dL)	1.47 (0.99-2.17)	0.053	0.77 (0.49-1.20)	0.248

The multivariate Cox regression analysis was adjusted for age, race, stroke, BMI, diastolic BP, hemoglobin, triglyceride, albumin, eGFR, serum sodium, serum potassium, serum chloride, prescription of beta-blockers, prescription of MRA, prescription of diuretics, and prescription of urate-lowering agents

Abbreviation: BMI, body mass index; BP, blood pressure, eGFR, the rate of estimated glomerular filtration rate; MRA, mineralocorticoid-receptor antagonist; HR, hazard ratio; CI, confidence interval

### RCS and threshold analysis

**Fig. 3:**
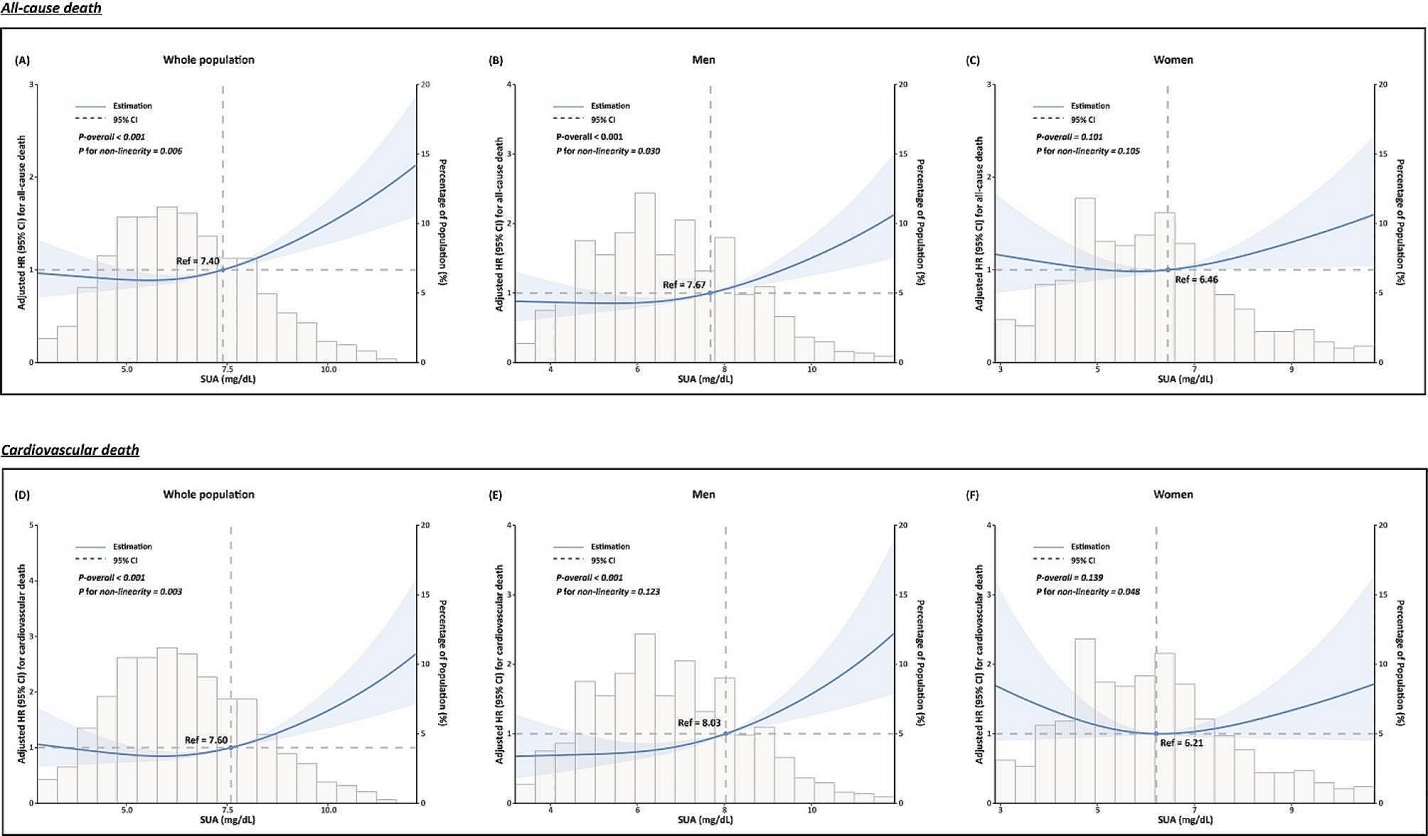
Dose-response relationship between SUA and all-cause mortality and cardiovascular mortality stratified by gender. (For all-cause mortality, covariates including, age, race, diabetes, stroke, drinking history, BMI, diastolic BP, WBC, hemoglobin, albumin, total cholesterol, eGFR, serum potassium, serum sodium, serum chloride, prescription of beta-blockers, prescription of MRA, prescription of diuretics, and prescription of urate-lowering agent, were adjusted. For cardiovascular mortality, covariates including age, race, stroke, BMI, diastolic BP, hemoglobin, triglyceride, albumin, eGFR, serum sodium, serum potassium, serum chloride, prescription of beta-blockers, prescription of MRA, prescription of diuretics, and prescription of urate-lowering agent, were adjusted.)

As shown in Fig. 3, RCS analysis of fully adjusted model revealed that SUA was associated with all-cause mortality and cardiovascular mortality in the whole population (*p* < 0.001, both). Furthermore, SUA had a non-linear relationship with all-cause mortality (*p* for nonlinearity = 0.006) as well as cardiovascular mortality for over-all population (*p* for nonlinearity = 0.003). When analyzed according to gender, the nonlinear relationship between SUA and all-cause mortality was only observed in male patients (*p* < 0.001, *p* for nonlinearity = 0.030), but not in female patients. For male patients, the risk of all-cause death showed an ascending trend as SUA levels increased, especially when SUA was higher than 7.67 mg/dL., Furthermore, there was a linear positive relationshipbetween SUA with cardiovascular mortality (*p* for nonlinearity = 0.123) for male patients. In contrast, no statistically significant relationship between SUA and all-cause and cardiovascular mortality was detected in female patients (*p* = 0.101, for all-cause mortality; *p* = 0.139, for cardiovascular mortality).

### Competitive risk analysis via Fine-Gray competing risk model

**Fig. 4:**
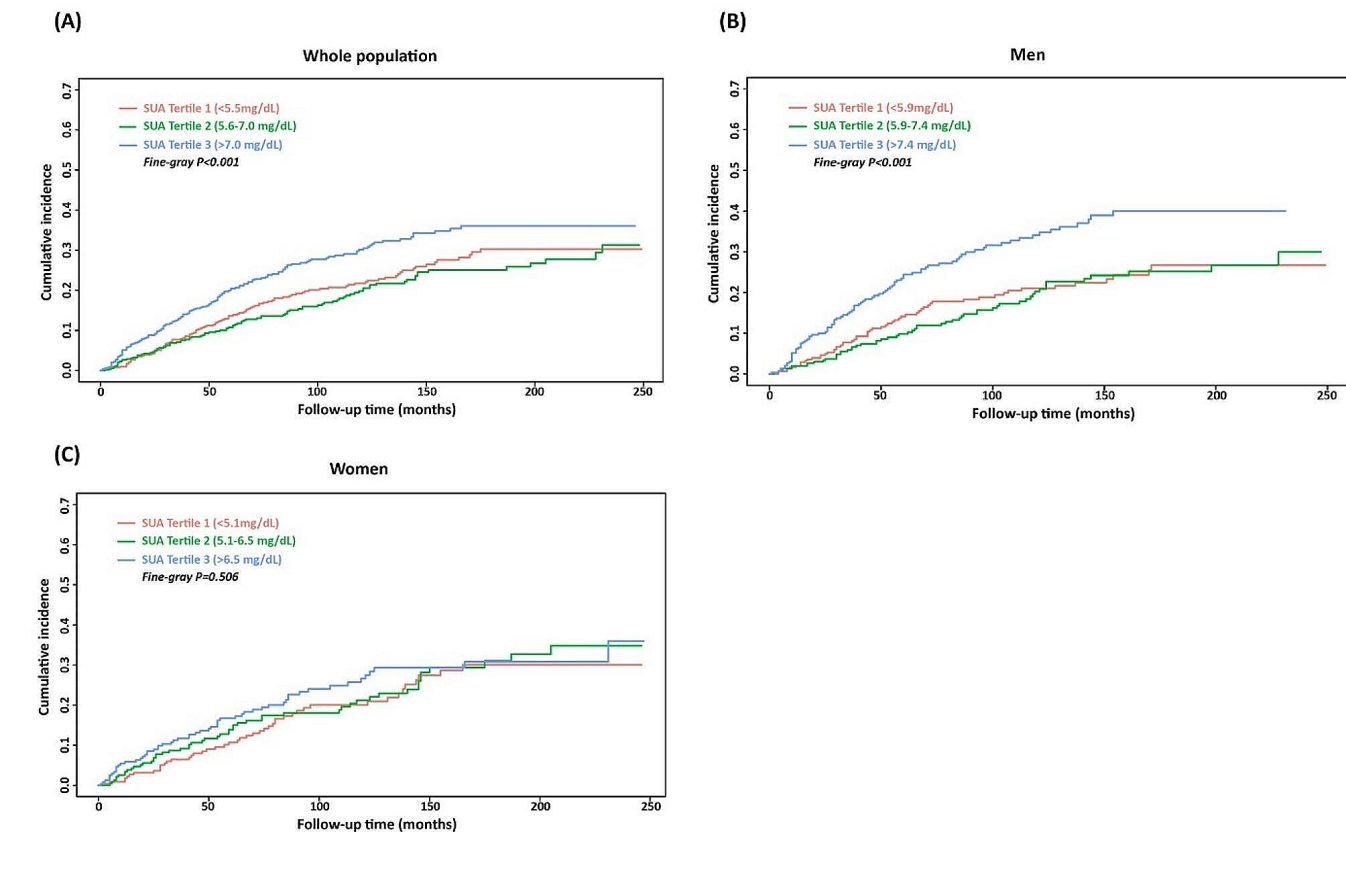
Association between SUA and cardiovascular deaths with non-cardiovascular death as a competing risk (Fine-Gray competing risk model)

To further explore the gender differences for the prognostic implication of SUA on cardiovascular mortality of CHF, a competing risk analysis via Fine-Gray competing risk model was performed, and non-cardiovascular death was used as a competing event. As illustrated in Fig. 4, Fine-Gray competing risk model test revealed that male patients with highest SUA tertile had highest cumulative risk of cardiovascular death than that of the other categories (Fine-Gray *p* < 0.001, Fig. 4B), while no significantly statistical difference was observed in female patients (Fine-Gray *p* = 0.506, Fig. 4C).

The results of the sub-distribution hazard function in the Fine-Gray model were shown in Table 6. For the whole population, univariable and multivariable analysis both demonstrated that SUA were significantly associated with cardiovascular mortality (SHR 1.14, 95% CI 1.08-1.21, *p* < 0.001, for univariable analysis; SHR 1.10, 95% CI 1.03-1.17, *p* = 0.006, for multivariable analysis). Furthermore, SUA performed well in prognostic discrimination of cardiovascular mortality for male patients (SHR 1.17, 95% CI 1.09-1.26, *p* < 0.001, for univariable analysis; SHR 1.17, 95% CI 1.08-1.27, *p* < 0.001, for multivariable analysis) while not for female patients (SHR 1.10, 95% CI 1.00-1.20, *p* = 0.044, for univariable analysis; SHR 0.98, 95% CI 0.87 − 1.10, *p* = 0.690, for multivariable analysis).

**Table 6:** Association of SUA and cardiovascular mortality via fine-gray competing risk model

	Non-adjusted model		Adjusted model	
	SHR (95% CI)	*p* value	SHR (95% CI)	*p* value
**Whole population (N=1593)**				
Continuous per 1 unit increase	1.14 (1.08-1.21)	<0.001	1.10 (1.03-1.17)	0.006
Tertile 1 (<5.5 mg/dL)	Reference	--	Reference	--
Tertile 2 (5.5-7.0 mg/dL)	0.94 (0.82-1.08)	0.370	0.94 (0.82-1.08)	0.410
Tertile 3 (>7.0 mg/dL)	1.12 (1.03-1.21)	0.007	1.06 (0.97-1.17)	0.200
**Men (N=897, 56.3%)**				
Continuous per 1 unit increase	1.17 (1.09-1.26)	<0.001	1.17 (1.08-1.27)	<0.001
Tertile 1 (<5.9 mg/dL)	Reference	--	Reference	--
Tertile 2 (5.9-7.4 mg/dL)	0.97 (0.81-1.16)	0.740	1.01 (0.84-1.22)	0.900
Tertile 3 (>7.4 mg/dL)	1.21 (1.09-1.35)	<0.001	1.19 (1.05-1.35)	0.006
**Women (N=696, 43.7%)**				
Continuous per 1 unit increase	1.10 (1.00-1.20)	0.044	0.98 (0.87-1.10)	0.690
Tertile 1 (<5.1 mg/dL)	Reference	--	Reference	--
Tertile 2 (5.1-6.5 mg/dL)	1.05 (0.87-1.28)	0.590	0.93 (0.75-1.14)	0.460
Tertile 3 (>6.5 mg/dL)	1.08 (0.95-1.22)	0.260	0.90 (0.77-1.06)	0.200

The adjusted model was adjusted for age, race, smoking history, diastolic BP, BMI, hemoglobin, albumin, eGFR, serum chloride, prescription of beta-blockers, prescription of MRA, prescription of diuretics, and prescription of urate-lowering agents

Abbreviation: BMI, body mass index; BP, blood pressure, eGFR, the rate of estimated glomerular filtration rate; MRA, mineralocorticoid-receptor antagonist; SHR, the sub-distribution hazard ratio; CI, confidence interval

## Discussion

According to our post-hoc analysis of the NHANES data, we found a significant gender-specific association between SUA and the long-term prognosis of HF in American populations. SUA was independently associated with all-cause mortality and cardiovascular mortality in men but not in women. The impact of gender difference on the role of SUA in the long-term prognosis discrimination in patients with HF has not previously been fully elucidated. To our knowledge, this is the first study to comprehensively describe the gender difference in the relationship between SUA and the long-term prognosis, especially for cardiovascular mortality, in patients with HF.

SUA is an important pathogenic risk factor for numerous cardiovascular diseases. Previous literatures have demonstrated that hyperuricemia or increased SUA levels were correlated with aggravated cardiometabolic risk, increased disease susceptibility, and poor prognosis in patients with coronary heart disease, atrial fibrillation, or HF [21–23]. SUA showed excellent prognostic discrimination value across all phenotypes and across the whole spectrum of ejection fraction subtypes of HF [6]. Carnicelli et al. revealed that SUA is an important biomarker of comorbidities and myocardial remodeling in patients with HF with preserved ejection fraction (HFpEF) [24]. For patients with HF with reduced ejection fraction (HFrEF), a sub-analysis of the EMPEROR-Reduced trial also identified hyperuricemia as a vital predictor of advanced disease severity and adverse prognosis [25]. Furthermore, HF with mid-range ejection fraction (HFmrEF) also exhibited increased risk of cardiovascular death and HF rehospitalization [26, 27]. Consistent with the findings of previous investigations, we found SUA was associated with increased all-cause mortality (HR 1.09, 95% CI 1.05-1.14) and cardiovascular mortality (HR 1.12, 95% CI 1.05-1.19), even after potential confounders. Overall, SUA is an important risk and prognostic biomarker for HF.

Gender differences in the association between SUA and cardiovascular outcomes have been reported for several cardiovascular diseases. For the majority of cardiovascular cases, SUA exhibited superior prognostic value in female patients compared with male patients. For patients with coronary heart disease, there was a positive correlation between SUA and disease severity and mortality [15, 28, 29]. For atrial fibrillation and metabolic syndrome, the pathogenic role of SUA is stronger in female patients than that in male patients [30, 31]. However, there are some controversies about the relationship between SUA and LVH. A pooled analysis revealed that hyperuricemia was associated with increased left ventricular mass index and LVH in women but not in men [17]. While for elderly patients with nonvalvular atrial fibrillation, SUA was identified as a significant risk factor for LVH in men only [32]. Furthermore, the correlation between SUA and arterial stiffness was more pronounced in men than in women among patients with hypertensive chronic kidney disease [33]. The prognostic value of SUA is also greater in male patients with non-obstructive coronary artery disease than in female patients [34]. Furthermore, the Gubbio population study, an epidemiological longitudinal survey based on a residential cohort, revealed that higher sex-specific SUA quintiles were associated with higher risk of short-term and long-term cardiovascular atherosclerotic events [35]. Gender difference of SUA is common and important in various cardiovascular diseases.

For the gender difference of SUA in HF, related studies are few and fragmentary. Previous studies had revealed gender specific relationship between SUA and fetal chronic HF (CHF) in general Austrian population. The Vorarlberg Health Monitoring and Promotion Program had demonstrated that SUA was an independent risk factor for fetal CHF for both men (aHR1.10, 95% CI 1.01–1.20) and women (aHR 1.10, 95% CI 1.00–1.20) [36–37]. However, in their adjusted model, renal function and eGFR, the important confounders of SUA, were not enrolled in their analysis, which may influence the interpretation of their conclusion. Another isolated post-hoc analysis of the Norwegian Heart Failure Registry reported the gender difference in the association between SUA and all-cause mortality in patients with chronic HF [38]. In contrast to our study, their research showed that elevated SUA levels were independently associated with 5-year all-cause mortality in women, but not in men. There are several defects in their study design and statistical analysis methodology. First, their conclusion was drawn by adopting SUA as a categorical variable, and their statistical significance was calculated by comparing SUA quartiles 1–3 to quartile 4. The P-trend largely depends on the selection of truncation values and how the subgroups were categorized. The dose-response relationship between SUA and the hazard ratio of all-cause mortality is more intuitive and reliable when SUA is analyzed as a continuous variable. Just like our study, although SUA was obvious in its association with cardiovascular mortality for the whole population when SUA was analyzed as a continuous variate, the P for trend had no statistical significance via analysis according SUA tertiles. Furthermore, their propensity score method was not rigorous. Their propensity score matching was conducted only for the whole population. When gender-specific analysis was performed, covariates may not be fully matched. Moreover, the sex difference of the association between SUA concentrations and cardiovascular mortality of HF patients was not addressed in their study. In another sub-analysis of the EVEREST trail, gender did not influence the association between SUA and clinical outcomes (including cardiovascular mortality and HF rehospitalization) in patients hospitalized for worsening HFrEF [39]. In our study, we revealed that the association between SUA and long-term prognosis was more pronounced in male HF patients than in female HF patients. For male patients with HF, the risk of all-cause death increased 11% (HR 1.11, 95% CI 1.05-1.18), and the risk of cardiovascular death increased 18% (HR 1.18, 95% CI 1.09-1.28), for per 1 mg/dL increase in SUA. The different clinical characteristics of participants in different trials may explain the discrepancies between our findings to those of previous literature. Our study provides additional insights into the gender difference of SUA in patients with cardiovascular diseases, especially for patients with HF.

For patients with hyperuricemia and gout, the age-standardized incidence of congestive HF is higher in men than that in women [40]. Furthermore, hyperuricemia is more prevalent in male HF patients than in female patients [41]. From this perspective, SUA is more important in male HF patients. Furthermore, previous clinical evidences provide additional supporting to our findings. For patients with HFrEF, the progressive increase in SUA was more obvious in men than women during one-year follow-up [42]. Borghi et al. reported that the negative relationship between SUA and left ventricular ejection fraction, the important indicator of the prognosis of HF, was observed only in male HF patients, but not in the female [43, 44]. Another study revealed that the positive association between SUA and left ventricular mass index (LVMI) existed only in men, but not in women. Based on the evidences of indicators of myocardial remodeling and left ventricular recovery, SUA was associated with poor prognosis in men. However, for the gender differences in the association between SUA and prognosis of HF, the physio-pathologic mechanism is still unclear. Further molecular biology experiments should be conducted.

### Study limitations

There are still several limitations in our study. Firstly, our study was a post-hoc analysis design of a national perspective study. We could only extract pre-existing covariates in the NHANES database for our analysis. The other important variables, such as echocardiographic parameters, may influence the interpretation of our results. Secondly, the diagnosis of HF in our analysis was based on the self-reported HF of participants, which was not entirely reliable. Thirdly, the sub-phenotype and severity of HF was not specifically analyzed. Finally, the conclusions were derived from the American population, essential further research should be conducted to extrapolate our findings to other racial and demographic populations.

## Conclusions

Gender differences in the association between SUA and the long-term prognosis in HF patients were nonnegligible. SUA was an independent predictor for all-cause mortality and cardiovascular mortality in men but not in women, even after adjustment for potential confounders and competing risk.

## Data Availability

No datasets were generated or analysed during the current study.
